# High fat diet drives obesity regardless the composition of gut microbiota in mice

**DOI:** 10.1038/srep32484

**Published:** 2016-08-31

**Authors:** Sylvie Rabot, Mathieu Membrez, Florence Blancher, Bernard Berger, Déborah Moine, Lutz Krause, Rodrigo Bibiloni, Aurélia Bruneau, Philippe Gérard, Jay Siddharth, Christian L. Lauber, Chieh Jason Chou

**Affiliations:** 1 Micalis Institute, INRA, AgroParisTech, Université Paris-Saclay, Jouy-en-Josas, France; 2 Nestlé Research Centre, Lausanne, Switzerland; 3 Nestlé Institute of Health Sciences, Lausanne, Switzerland

## Abstract

The gut microbiota is involved in many aspects of host physiology but its role in body weight and glucose metabolism remains unclear. Here we studied the compositional changes of gut microbiota in diet-induced obesity mice that were conventionally raised or received microbiota transplantation. In conventional mice, the diversity of the faecal microbiota was weakly associated with 1^st^ week weight gain but transferring the microbiota of mice with contrasting weight gain to germfree mice did not change obesity development or feed efficiency of recipients regardless whether the microbiota was taken before or after 10 weeks high fat (HF) feeding. Interestingly, HF-induced glucose intolerance was influenced by microbiota inoculation and improved glucose tolerance was associated with a low Firmicutes to Bacteroidetes ratio. Transplantation of Bacteroidetes rich microbiota compared to a control microbiota ameliorated glucose intolerance caused by HF feeding. Altogether, our results demonstrate that gut microbiota is involved in the regulation of glucose metabolism and the abundance of Bacteroidetes significantly modulates HF-induced glucose intolerance but has limited impact on obesity in mice. Our results suggest that gut microbiota is a part of complex aetiology of insulin resistance syndrome, individual microbiota composition may cause phenotypic variation associated with HF feeding in mice.

Macro- and micro-nutrients provide energy and essential building blocks for growth and maintenance of our body. Excessive over and under nutrition causes obesity and Kwashiorkor, respectively, conditions that affect millions of people in many developed and developing countries. Interestingly, both obesity and Kwashiorkor are associated with modifications of faecal microbiota, indicating a close relationship between nutrition availability and faecal microbiota composition^1^,^2^. Transplantation of faecal microbiota from undernourished Malawian children compared to that of healthy children into germfree mice showed that mice with microbiota of poorly nourished children had impaired growth when fed a nutrient poor diet^3^. This result highlights the importance of a healthy microbiota to support early stages of development, especially under a compromised nutritional condition. Not only can gut microbiota influence the nutritional value of the diet, nutrition can shape the composition of faecal microbiota. Wu *et al*. demonstrated that faecal microbiota rapidly respond to dietary changes in a controlled feeding experiment in healthy subjects^4^, and the response of microbiota to a diet change is as short as 1 day in humans^5^.

Earlier evidence showed that faecal bacterial communities low in Bacteroidetes are associated with human obesity^1^ suggesting a possible connection between microbes and health. This finding, however, has been confirmed in some studies^6^,^7^ but rejected in others^8^,^9^. The underlying mechanisms for obesity are multi-factorial, and thus it is not surprising to see inconsistent relationships between certain bacterial groups and body weight being reported in the literature as age, geographical location, and diet have all been shown to contribute to the variability of faecal microbiota composition in mammals^4^,^10^.

Recent twin studies have advanced our knowledge about the relative importance of host genetics and gut microbiota in obesity. These studies showed that faecal microbiota are more similar among twins than unrelated individuals^7^,^11^, supporting the notion that host genetics is a considerable factor on faecal microbial composition. A similar conclusion was obtained when comparing the microbiota of 8 different mouse lines^12^. Mice with the same genetic background had a more similar microbiota composition than mice with different background. The genotype specific gut microbiota can be an important factor contributing to phenotypic characteristics of the host. For example, TLR5 knockout mice showed a distinguished gut microbiota from the wild type controls, and transplantation of the TLR5 knockout microbiota to germfree mice demonstrated the causal role of microbiota in metabolic regulation of the host^13^. However, this does not explain whether gut microbiota influence the overall physiology of the host independent of genetic background. In animals, nearly genetically identical inbred C57BL/6J mice exhibit inter-individual variations in body weight when consuming a high fat (HF) diet^14^,^15^, but the cause for inter-individual variability remains elusive. Fan *et al*. showed that epigenetic markers are associated with diet-induced obesity (DIO) and many differentially methylated genes are involved in lipid metabolism and immunity^16^. Nevertheless, the near homogeneous genetic background DIO mice represents an interesting model for directly studying the relationship between body weight variation and microbiota composition.

To answer whether gut microbiota composition is a critical factor for HF diet induced obesity, we performed several microbiota transplantation experiments in mice. By transplanting faecal microbiota from selected donors to axenic, inbred, germfree C57BL/6J mice, it allowed us to study the importance of gut microbiota to the development of obesity in an identical genetic background and nutritional condition. Here we hypothesized that heterogeneous body weight resulting from HF feeding can be attributed to the composition of microbiota. To test this hypothesis, we first measured the faecal microbiota prior to and after HF feeding in mice. Second, we transplanted the pre- and post-HF feeding faecal microbiota to germfree mice and monitored the development of obesity and food intake under the HF feeding condition. Since HF feeding also induces insulin resistance in C57BL/6J mice, we measured glucose tolerance of the animals to ascertain the role of gut microbiota in glucose metabolism of the host.

## Results

### Individual microbiota does not dispose mice to diet-induced obesity

**Figure 1:**
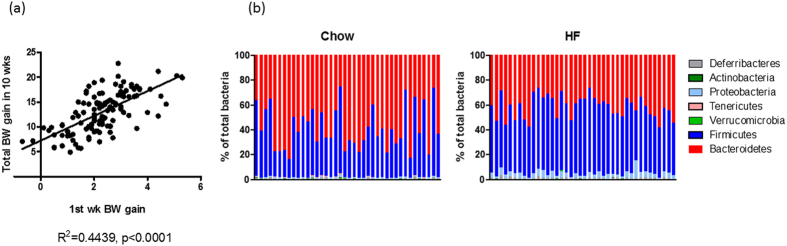
Relationship of body weight and microbiota in diet induced obesity (DIO) mice. (**a**) Correlation of 1^st^ week weight gain and total weight gain after 10 week high fat diet feeding in 149 mice. (**b**) Faecal microbiota of before and after 10 weeks high fat feeding. Baseline faecal samples were collected from 40 DIO mice when fed a chow diet. Samples were also collected from the same mice after 10 weeks high fat feeding. Microbiota composition was analysed with 16S rDNA sequencing and the abundances of major phyla are shown.

HF feeding induces obesity but individual responses differ tremendously. By examining the body weight evolution of 149 diet-induced obesity (DIO) mice over time, it is clear that the relationship between the 1st week weight gain and total weight gain at 10 wks was significant (Fig. 1a). This result suggests that factors prior to HF feeding or initial responses to HF feeding drive the development of obesity in mice. We compared faecal microbiota before (e.g. on chow) and after 10 weeks of HF feeding in 40 C57BL/6J mice by sequencing the V4 region of the 16S rDNA gene. Our results showed a high degree of inter-individual variability in both dietary conditions (Fig. 1b), and the ratio of Firmicutes to Bacteroidetes (two most dominant bacteria phyla expressed as (F/B)) ranged from 0.18 to 2.73 on chow and 0.69 to 2.56 on HF, respectively. We performed regression analysis to examine the relationship between acute and chronic body weight gain of individuals to faecal microbiota α-diversity prior to HF feeding. Our data showed a significant relationship between the first week weight gain with Shannon and Chao 1 diversity indices whereas only a trend with Inverse Simpson and no relationship with microbiota F/B ratio were found (Table 1). However, none of the diversity indices had a significant relationship with total body weight gain (Table 1). At the family level, the abundance of Clostridiales Incertae Sedis XIII negatively correlated with 1^st^ week weight gain (p = 0.0235, r = −0.357) and total weight gain of mice on HF diet (p = 0.0422, R = −0.323) but this is no longer statistically significant after Bonferroni correction. Relationships between α−diversity indices and final body weight of DIO mice were also examined. Body weight positively correlated with Inverse Simpson and Shannon index but no relationship was found with Chao1 or F/B ratio (Table 1).

**Figure 2:**
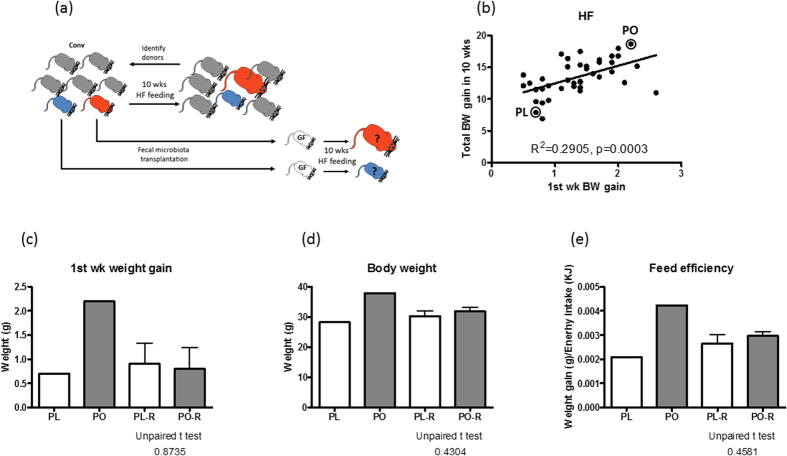
Transplantation of PO or PL microbiota does not change the body weight of mice. (**a**) Scheme of experimental design is shown. Faecal samples were collected from 40 mice before switching to a high fat diet. Two microbiota donors were selected and their pre-high fat feeding faecal samples were transplanted to two groups of germfree mice. Microbiota recipients were challenged with a high fat diet for 10 weeks. (**b**) The criteria for selecting microbiota donors is based on 1^st^ week and total body weight gain. Two donors, pre-lean (PL) and pre-obese (PO) and their weight gain performance in relation to the rest of mice are shown in a correlation graph. Comparison between donors and microbiota recipients in 1^st^ week gain (**c**), final body weight (**d**) and feed efficiency (**e**) are shown. Unpaired t-tests were perform to compare PL-R and PO-R mice. Data are mean ± s.e.m., n = 12/group.

Next, we examined if the gut microbiota prior to HF feeding could influence body weight of mice on HF diet by conducting a faecal microbiota transplantation experiment. The design of the experiment is illustrated in Fig. 2a. Faeces of 40 mice were collected prior to HF feeding and were stored at −80 **°**C. To select the microbiota donors, we screened DIO mice based on the 1^st^ week weight gain and 10 week weight gain, and identified two donors with contrasting weight gain parameters (Fig. 2b). Then, previously stored faecal samples of the donors, designated as pre-obese (PO) and pre-lean (PL), were transplanted into germfree mice and housed in isolators with free access to a HF diet for 10 weeks. After one week of HF feeding, PO microbiota receiving (PO-R) and PL microbiota receiving mice (PL-R) gained a similar amount of weight, a characteristic different to their donors (Fig. 2c). Also unlike the donors, final body weight as well as the feed efficiency, defined as body weight gain divided by energy intake, were indistinguishable between PL-R and PO-R (Fig. 2d,e).

**Figure 3:**
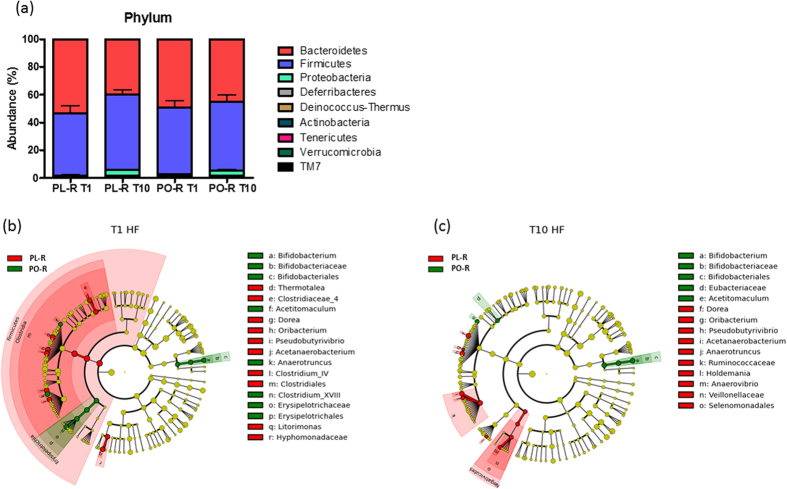
Microbiota composition of PL-R and PO-R mice (**a**) Major faecal bacteria phyla of the PL-R and PO-R mice are shown. Faecal samples were collected at one week and 10 after microbiota transplantation. After the transplantation, mice were placed on a HF diet. Comparison of faecal microbiota was performed with LDA effect size (LEfSe). The red area indicates the over-abundance in PL-R microbiota where the green area indicated the over-abundance in PO-R microbiota. The difference between PL-R and PO-R microbiota at 1 week after (**b**) and 10 week after (**c**) HF feeding are shown.

Faecal microbiota of the PO-R and PL-R mice at 1 week and 10 weeks post faecal transplantation were analysed and our results showed that Firmicutes and Bacteroidetes were the two dominant bacterial phyla in all mice (Fig. 3a). When comparing the microbiota of PL-R and PO-R mice using LDA effect size (LEfSe) calculation, many taxa were found to be different in abundance at week 1. The Firmicutes were most abundant in PL-R mice compared to PO-R mice that had greater abundances of Erysipelotricaceae and *Bifidobacterium* (Fig. 3b and Supporting Figure S1). After 10 weeks of HF feeding, microbiota between PO-R and PL-R mice became more homogenous than 1 week, with only a few taxa remaining significantly different between the two groups. Notably, bacteria belonging to the Firmicutes such as *Oribacteriaum*, *Acetanaerobacterium*, *Dorea*, *Pseudobutyrivibrio* and Ruminococcaceae were more abundant in PL-R, whereas *Acetitomaculum* and *Bifidobacterium* were consistently more abundant in PO-R mice (Fig. 3c and Supporting Figure S2). Our data indicate that HF feeding strongly affected the composition of the gut microbiota but that some taxa resisted HF feeding, and the initial differences between the two microbiota persisted till the end of the HF challenge (Fig. 3b,c). However, dissimilarities between the PL-R and PO-R taxa were not related to body weight nor feed efficiency of DIO mice.

### Transplantation of microbiota resulting from HF feeding caused similar weight gain irrespective of donors

**Figure 4:**
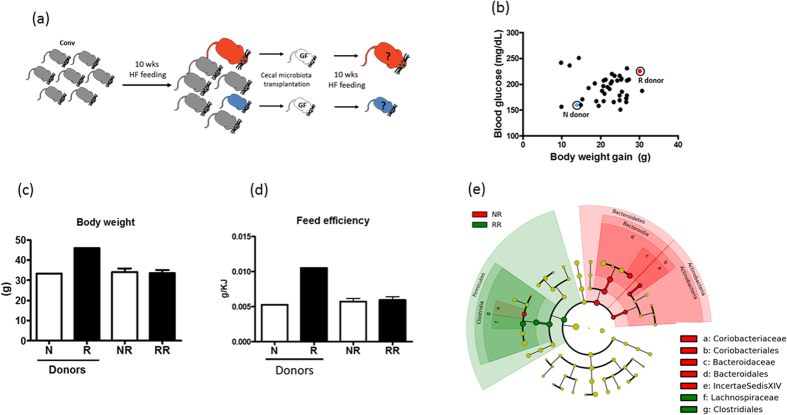
Transplantation of post-high fat feeding microbiota has no effect on body weight of mice. (**a**) The design of the experiment is illustrated. Briefly, mice were challenged with a HF diet for 10 weeks and a responder (R) and non-responder (N) were selected as microbiota donors. The caecal microbiota was transplanted to two groups of germfree mice and recipients were fed the same high fat diet for 10 weeks. (**b**) Selection of donors was based on total weight gain and 6 hour fasting blood glucose concentration. The parameters of the donors are indicated in a correlation graph indicating the opposite physiological characteristics of the donors. Body weight (**c**) and feed efficiency (**d**) of microbiota donor and recipients (NR and RR) are shown. Unpaired t-tests were perform to compare PL-R and PO-R mice. Data are mean ± s.e.m., n = 12/group. Comparison of NR and RR microbiota after 10 weeks HF feeding was performed using LDA effect size (LEfSe). The red area indicates the over-abundance in NR microbiota where the green area indicated the over-abundance in RR microbiota (**e**).

To further examine the relationship between gut microbiota and diet-induced obesity, we performed a second transplantation experiment using the caecal microbiota of conventional mice post HF feeding as inocula for germfree mice (Fig. 4a). Donors were selected based on body weight and fasting glycemia level after being on a HF diet for 10 weeks. The caecal contents of an obese (46.0 g) and severely hyperglycemic (223 mg/dL) responder (R) and a less obese (33.1 g) and mildly hyperglycemic (158 mg/dL) non-responder (N) were transplanted to germfree mice by oral gavage (Fig. 4b). After the transplantation, all recipients were maintained in isolators and were fed a HF diet for 10 weeks. Body weight and food intake were recorded weekly. Shown in Fig. 4c, despite the 12.9 g difference in body weight between the N and R donors, their corresponding recipients (NR and RR, respectively) were not significantly different in body weight after 10 weeks (Fig. 4c). Feed efficiency was also indistinguishable between NR and RR mice unlike their donors (Fig. 4d). Comparison of caecal microbiota revealed that microbiota of the NR significantly differed from that of RR mice (Fig. 4e). NR caecal microbiota were dominated by Bacteroidetes (order Bacteroidales and family Bacteroidaceae) and Actinobacteria (order Coriobacteriales and family Coriobacteriaceae), whereas RR caecal microbiota were enriched with Firmicutes (order Clostridiales and family Lachnospiraceae, Fig. 4e).

### High abundance of Bacteroidetes correlated with improved glucose tolerance in DIO mice

**Figure 5:**
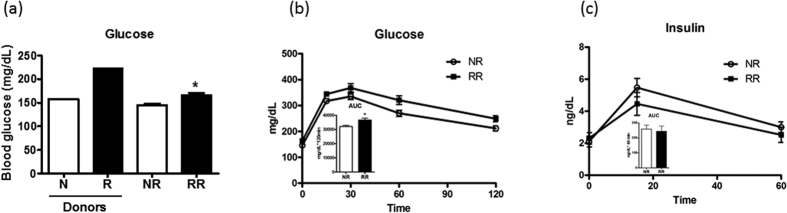
Transplantation of microbiota sufficiently altered the glucose metabolism of the recipients. (**a**) 6 hours fasting blood glucose concentrations of donors (N and R) and recipients (NR and RR) are shown. (**b**) Oral glucose tolerance test (OGTT) was performed near the end of HF feeding period. Blood glucose taken at the baseline, 15, 30, 60 and 120 min after the oral glucose gavage are shown. Insert shows the area under the glucose curve. (**c**) Blood samples were taken from the tail vein at baseline, 15 min and 60 min after oral glucose gavage, and plasma insulin concentrations were determined. Insert indicates the area under the insulin curve. Data are mean ± s.e.m., n = 12/group. *p < 0.05 by unpaired t-test.

Interestingly, NR mice had lower fasting glycaemia than RR mice, a relationship that is similar to their donors (Fig. 5a). In addition, NR mice were more glucose tolerant than RR mice (Fig. 5b), and the difference in glucose tolerance was not due to insulin as the concentrations during the OGTT were indistinguishable (Fig. 5c). However, differential glucose tolerance between NR and RR mice was associated with contrasting microbial profile with Bacteroidetes being the dominant phylum in NR microbiota whereas RR was dominated by Firmicutes (Fig. 4e). To further investigate the relationship between glucose tolerance and gut microbiota composition, we compared the overall composition of the microbiota and the oral glucose tolerance of PL-R and PO-R mice. Between PL-R and PO-R mice, the β-diversity of gut microbiota calculated with ThetaYc index near the time of oral glucose tolerance test differed significantly (AMOVA, p < 0.001), and many bacteria belonging to the Firmicutes such as *Acetanaerobacterium*, *Dorea*, *Pseudobutyrivibrio*, and Ruminococcaceae were significantly higher in PL-R than in PO-R (Fig. 3c and Supporting Figure S2). Since many Firmicutes were more abundant in PL-R, we postulated that PL-R would be more glucose intolerant than PO-R mice. Indeed, PL-R had a trend of higher glucose excursion during the OGTT than PO-R (p = 0.0726, Supporting Figure S3). Overall, the results demonstrate that different microbiota resulting from HF feeding significantly influenced the glucose metabolism of mice, and communities dominated by Bacteroidetes showed a protective effect against HF-feeding induced glucose intolerance.

**Figure 6:**
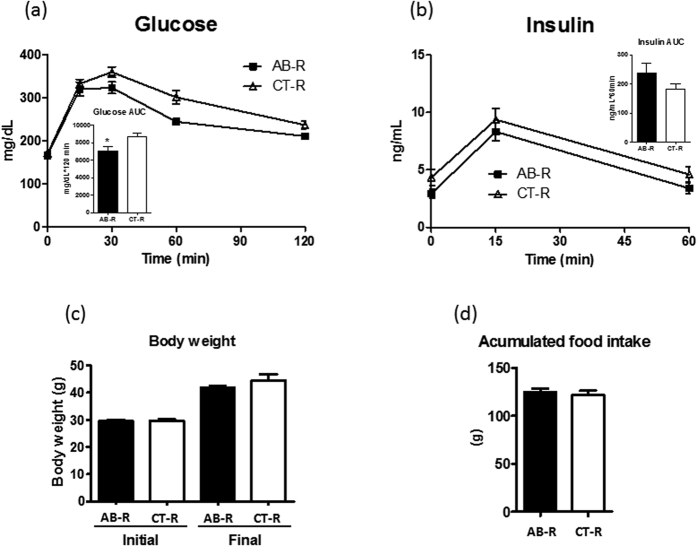
Antibiotic modified microbiota enhances glucose tolerance of DIO mice. Germfree mice were colonized with either antibiotic treated (AB) or untreated control (CT) microbiota. A combination of polymyxin B and neomycin was added to drinking water of DIO mice and caecal microbiota of a treated and untreated mouse were selected as microbiota donor. The microbiota recipients (AB-R and CT-R) were challenged with HF feeding and oral glucose tolerance was evaluated at week 7. Blood glucose (**a**) and plasma insulin (**b**) concentrations are shown and inserts are the area under glucose or insulin curve, respectively. Body weight and accumulated food intake of donors and recipients are shown in (**c,d**). Data are mean ± s.e.m., n = 12/group. *p < 0.05 by unpaired t-test.

Previously, we found that treating DIO mice with polymyxin B and neomycin in drinking water changed the caecal microbiota towards a Bacteroidetes dominated profile (Supporting Figure S4) and improved glucose tolerance when compared to untreated mice^17^. This suggests that Bacteroidetes rich communities provide a protective effect on blood glucose level, but the use of antibiotics could impact the outcome independent of microbiota modification. To verify that Bacteroidetes rich microbiota resulting from the antibiotic treatment drives the improvement of glucose metabolism, we transplanted the caecal contents from antibiotic treated (polymyxin B 1.0 g/L and neomycin 0.5 g/L in drinking water) or untreated mice to germfree mice. Mice were kept in isolators and were fed a HF diet for 7 weeks before an oral glucose tolerance test was administered. DIO mice colonized with antibiotic associated (AB-R) microbiota were more glucose tolerant than mice colonized with normal control (CT-R) microbiota, a difference that could not be accounted for via plasma insulin concentrations (Fig. 6a,b). More importantly, both body weight and food intake were indistinguishable between the groups (Fig. 6c,d) at the end of seven weeks.

**Figure 7:**
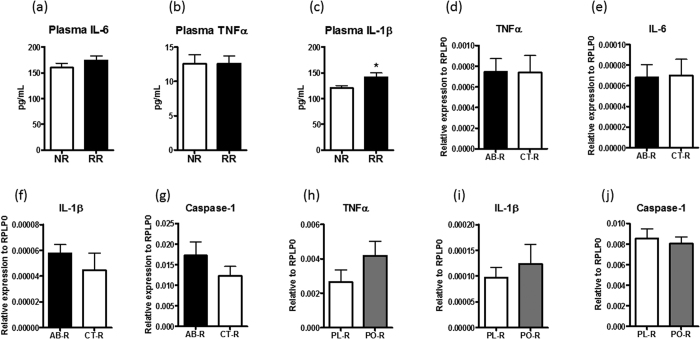
Inflammatory status of mice with different microbiota background. Proinflammatory cytokine levels in the plasma of NR and RR mice were measured at the end of the study and the concentrations of IL-6 (**a**), TNFα (**b**) and IL-1β (**c**) are shown. The mRNA of TNFα (**d**), IL-6 (**e**), IL-1β (**f**) and Caspase-1 (**g**) in the epididymal adipose tissue of AB-R and CT-R were determined with real time quantitative PCR method. The mRNA expression of TNFα (**h**), IL-1β (**i**) and Caspase-1 (**j**) were also measured in the epididymal adipose tissue of PL-R and PO-R mice. Data are mean ± s.e.m., n = 8–12/group. *p < 0.05 by unpaired t-test.

As HF feeding-induced insulin resistance is often associated with low grade inflammation in this animal model, we measured circulating proinflammatory cytokines to estimate the degree of immune response. Circulating IL-6 and TNFα concentrations were similar in RR and NR mice (Fig. 7a,b), whereas IL-1β was slightly but significantly elevated in RR mice (Fig. 7c). To better understand whether tissue inflammation can explain the differential glucose tolerance of mice with different F/B microbiota, we examined the mRNA expression of IL-17, IL-22, TNFα, IL-6, IL-1β and Caspase-1 in the epididymal adipose tissue of other studies. Between AB-R and CT-R mice, TNFα, IL-6, IL-1β and caspase-1 expressions were similar (Fig. 7d–g). IL-6 was undetectable and no difference was found in TNFα, IL-1β and Caspase-1 mRNA when comparing the epididymal adipose tissue of PL-R and PO-R mice (Fig. 7h–j). In addition, IL-17 and IL-22 mRNA was also undetectable in the epididymal adipose tissue in both studies. Based on our findings, microbiota contributed to diet-induced glucose tolerance but this did not cause significant changes in the expression of proinflammatory genes in the adipose tissue.

## Discussion

The most important finding of the present study is the limited role of microbiota in HF diet induced obesity in mice. We examined the microbiota composition of mice and did not find a clear relationship linking the F/B ratio of faecal microbiota to diet induced obesity, a finding confirmed by two independent microbiota transplantation experiments. Regardless of whether the microbiota were taken from mice before or after the HF feeding, the development of obesity was independent of the donor’s faecal/caecal microbiota profile or body weight.

Early work led by us and others showed that germfree mice remain lean and glucose tolerant on a HF diet^18^,^19^. This suggests that gut microbes are important for the development of DIO phenotypes. The current study extends our knowledge and reveals that obesity resulting from HF feeding was unrelated to the microbial composition of conventional and gnotobiotic mice. However, our results are in contrast to other studies that show microbiota composition is a determinative factor of body weight in mice fed diets rich in plant fibres^20^,^21^,^22^ or saturated fat^23^. The inconsistent results can be due to unique experimental design, microbiota, nutrition, and diet to each study. In particular dietary fibre likely plays a critical role in the relationship between gut microbiota and obesity and is supported by the observation that additions of psyllium or sugar cane fibre to a cellulose-based HF diet can reduce weight gain^24^. Even the type of fat fed to mice can have profound impact on body mass and gut microbiota where mice fed unsaturated fat gained less weight and harboured more diverse microbiota compared to those fed a diet high in saturated fat^25^. These studies demonstrated that diet quality is critical in determining gut microbiota compositions and phenotypic outcomes. Genetic background of mice can be another factor influencing the outcomes of our study. C57BL/6J mice are known to develop obesity on a HF diet^26^, and chronic exposure to high saturated fat can lead to an experimental condition that strengthens diet-host interactions while limiting the role of the gut microbiota in the development of obesity.

Interestingly, the abundance of faecal Clostridiales Incertae Sedis XIII prior to HF feeding was negatively correlated with 1^st^ week and total weight gain of mice on HF diet. Clostridiales Incertae Sedis XIII is a poorly characterized bacterial family, the potential function of which is largely unknown^27^. In our dataset, *Anaerovorax* is the most abundant genus in Clostridiales Incertae Sedis XIII family. In the literature, *Anaerovorax odorimutans*, was shown to produce butyrate by metabolizing putrescine^28^. The benefit of butyrate for diabetes is well-documented in studies showing the suppression of HF induced obesity after sodium butyrate supplementation^29^,^30^. Although this can explain the negative correlation between Clostridiales Incertae Sedis XIII and HF induced obesity in our study, the role of *Anaerovorax* in modulating weight gain on HF feeding is uncertain. Future work is required to dissect the direct and indirect effect of *Anaerovorax* on body weight regulation. Specifically, it is critical to determine whether the variation of body weight gain among individuals directly depends on the abundance of *Anaerovorax* in the gut or to the amount of butyrate produced by bacterial fermentation. Moreover, it is also necessary to understand how dietary proteins and amino acids act as a substrate for butyrate production by *Anaerovorax* and whether the resulting butyrate from amino acid fermentation plays a role in weight gain under a HF feeding condition.

Another important finding of our study is that gut microbiota rich in Bacteroidetes protect mice from developing severe glucose intolerance in a HF feeding condition. In the literature, low F/B ratios are associated with reduced blood glucose levels or increased glucose tolerance^20^,^31^, but our results extended this evidence by demonstrating the protective role of a low F/B microbiota to HF induced glucose intolerance in several faecal transplantation experiments. First, a clear distinction between the caecal microbiota composition and the degree of glucose tolerance was found in NR and RR mice. NR mice were high in Bacteroidetes and low in Firmicutes and showed reduced glucose excursion during glucose tolerance test compared to RR mice. Also, fasting blood glucose levels of microbiota recipients had a similar trend to their donors. Second, PL-R mice with increased faecal Firmicutes were more glucose intolerant compared to PO-R mice. The abundant Firmicutes found in PL-R faecal microbiota include many bacterial families such as Erysipelotrichaceae (*Holdemania*), Ruminococcaceae (*Anaerotruncus* and *Acetanaerobacterium*), Lachnospiraceae (*Oribacterium*, *Dorea* and *Pseudobutyrivibrio*) and Veillonellaceae (*Anaerovibrio*). Searching in Human Microbe-Disease Association Database (HMDAD) results in links to liver cirrhosis with *Holdemania* and *Dorea* and type 1 diabetes with *Anaerotruncus*, but not specific to insulin resistance that is commonly associated with HF feeding^32^. Since the involvement of bacteria at deeper taxonomical levels in glucose intolerance is uncertain, we focused on the phylum level distinction and examined whether F/B ratio of microbial communities is a factor in HF-induced glucose intolerance. In the last experiment, we demonstrated that transplantation of a low F/B caecal microbiota to germfree mice reduced the development of HF-induced glucose intolerance of ex-germfree mice. Altogether, results of three transplantation experiments showed that glucose intolerance related to HF feeding can be mitigated by microbiota enriched in Bacteroidetes compared to Firmicutes, resulting in measurable and significant improvements in blood glucose concentration independent of body weight.

In addition to the F/B ratio, increased Bifidobacteriales was found in PO-R compared to PL-R mice. Many strains of *Bifidobacteria* have been shown to have an antidiabetic effect. In one study, six week intervention with *Bifidobacterium animalis spp. lactis* 420 at the dose of 10^9^ (cfu day^−1^) enhanced glucose tolerance and elevated glucose turnover rate in DIO mice^33^,^34^. In another study, a mixture of 4 different *Bifidobacterium spp* were administrated to HF fed Swiss-Webster mice and the improvement in glucose tolerance was observed^35^. As the inter-individual variation of gut microbiota is evident in this study, understanding the environmental factors and background microbiota associated with each mouse would be necessary to advance a probiotics-based intervention strategy for managing type 2 diabetes.

Despite the consistent findings between low F/B ratio and resistance of glucose intolerance in DIO mice, it is not clear which host mechanisms affecting glucose metabolism are related to shifts in microbiota. In the literature, many pathways have been proposed to explain how the gut microbiota regulates glucose metabolism. For example, endotoxemia resulting from bacterial LPS induced inflammatory response has been associated with insulin resistance in DIO mice^36^,^37^. Although we did not measure the circulating LPS or LPS binding protein concentration in our studies, we observed that plasma IL-1β was lower in the NR compared to RR mice and the diminished IL-1β was associated with a lower excursion of glucose during OGTT. As IL-1β can also cause insulin resistance and beta cell toxicity, two aetiologies of type 2 diabetes, it is plausible that proinflammatory cytokines released by the intestine in response to different bacterial antigens affect glucose metabolism. However, not all insulin resistance-related proinflammatory cytokines were different in this study as TNFα and IL-6 were similar in NR and RR mice. Moreover, the mRNA expressions of pro-inflammatory cytokines were unchanged in the epididymal adipose tissue of the mice with different degree of glucose intolerance. This finding was consistent in the two microbiota transplantation experiments, namely PL-R/PO-R and AB-R and CT-R studies. Recently, Garidou *et al*. showed that mice with HF-induced diabetes have reduced IL17 + CD4 + T cells in the ileum, and this reduction is associated with changes in microbiota composition in the ileal mucosa^38^. The importance of ileal mucosa microbiota was later demonstrated in a microbiota transplantation experiment where the glucose tolerance of ex-germfree mice depends on whether or not the donor was previously treated with a combination of probiotics and prebiotics. This study highlighted the critical role of small intestinal microbiota in HF-induced insulin resistance in mice and open a new angle on how the ileal microbiota influences the differentiation of intestinal T cells as a pathway to regulate glucose homeostasis. Other mechanisms such as browning of white adipose tissue^39^, secretion of incretins^40^,^41^ and concentrations of short chain fatty acids^42^ have also been shown to affect glucose metabolism of the host. These inflammatory based pathways are credible candidates for future studies focusing on mechanisms by which communities with low F/B ratios regulate host glucose metabolism.

One of limitations of the current study is that the microbiota transplantation was performed with frozen stools of PL and PO mice. Due to the experimental design, we wouldn’t be able to inoculate fresh faecal microbiota because all the mice were on chow diet and their potential to develop diet-induced obesity was unknown at the time of faecal sample collection. In order to evaluate the possible impact of sample storage on the transmission efficiency, we performed a pilot study and compared the faecal microbiota of ex-germfree mice inoculated with a 100 fold diluted fresh feces or diluted faecal samples that were at −80 °C for 6, 17 and 29 weeks. The group colonized with the microbiota that was stored for 29 weeks showed an altered faecal microbiota with a marked increase of the *C. leptum* group. The groups receiving the frozen feces stored for 6 and 17 weeks had similar faecal microbiota composition to mice inoculated with fresh feces. The only exception is a reduction of faecal *Akkermansia muciniphila* in the group that received an inoculum from 17 weeks storage compared to the group that received fresh feces (fresh: 1.1 ± 0.7% vs 17 week: 0.2 ± 0.1%; mean ± SD, n = 6, p < 0.05). Based on the results of pilot study and the condition that PL and PO donors can be confidently identified after 10 weeks HF feeding, storage of PL and PO faecal samples was limited to 16 weeks. However, the reduction of *A. muciniphila* due to the sample storage might influence the outcomes of the study as Everard *et al*. reported that intestinal *A. muciniphila* has direct and positive impact on reducing high fat diet-induced glucose intolerance in mice^43^. To address this concern, we examined the abundance of *A. muciniphila* in the beginning and at the end of HF feeding. Based on 16S sequencing analysis, *A. muciniphila* was not detectable in all PL-R and PO-R faeces after 1 week HF feeding. At the end of HF period, a low abundance of *A. muciniphila* was detected in both groups but no statistical difference was found (PO-R: 2.45714E-06+/− 1.15487E-06; PL-R: 5.59E-06+/− 2.35E-06; mean+/− sem, n = 10–11). Although we cannot completely rule out the issue caused by sample storage, we did not find sufficient evidence suggesting the loss of *A. muciniphila* due to samples storage is the primary cause of differential glucose tolerance in PL-R and PO-R mice.

The HF diet induced obesity mouse model is widely used for studying the aetiology of insulin resistance and the development of novel therapeutics to treat type 2 diabetes. Here we demonstrate that the contribution of gut microbiota to obesity in C57BL/6J mice is very limited. However, the composition of the gut microbiota predispose mice for HF feeding induced glucose intolerance. In essence, our data extend the growing body of evidence that supports the host-microbe interactions as an integral part of metabolism by identifying gut microbiota as a physiologically relevant factor for previous unexplained phenotypic variations of HF feeding. In this respect, establishing gnotobiotic mice with defined microbiota would allow us to specify the host-microbes interactions in the context of searching for novel drug treatments and nutritional interventions for the treatment of insulin resistance.

## Methods

### Animals, diets, and tissue collection

Procedures were carried out in accordance with the European Guidelines for the Care and Use of Laboratory Animals and approved by the French Veterinary Authorities (Authorization number 78–58) or by “Office Vétérinaire Cantonal du canton de Vaud” Lausanne, Switzerland (Authorization number 1963). All conventional and germfree male C57BL/6J mice were purchased at 6 weeks of age from Charles River Laboratories (L’Arbresle, France) or were bred at Anaxem, the INRA germfree animal facility (Jouy-en-Josas, France). Upon arrival, mice were housed individually under a 12 h light/dark cycle for 2 weeks. All mice were given autoclaved water and γ-irradiated chow (45 kGy) or a HF diet (25 kGy, 60% energy from fat, D12492; Research Diets, New Brunswick, NJ). Mice were sacrificed by isoflurane overdose at the end of each study. Blood sample was collected from cardiac puncture and transferred to EDTA coated tubes and plasma samples were stored at −80 °C. Tissues were flash frozen in liquid nitrogen and stored at −80 °C.

For testing the PL and PO microbiota, faecal samples were collected before and after 10 weeks of HF feeding (60% energy from fat, D12492; Research Diets, New Brunswick, NJ) from conventionally raised mice and stored at −80 °C. Body weight was monitored during the HF feeding period and donors (PL, PO) were selected retrospectively based on the total and the first week weight gain. Frozen faecal pellets from each donor were resuspended (1/100 w/v) into a Ringer lactate buffer with hydrochloride L-cysteine (0.5 g/50 mL) and the faecal suspension was inoculated to 12 4 weeks old germfree mice by oral gavage. Mice with the same microbiota were housed individually in a single isolator, and fed a sterilized HF diet (γ-irradiation at 25 kGy, 60% energy from fat, D12492; Research Diets, New Brunswick, NJ) and water were ad libitum. Oral glucose tolerance tests were performed after 10 weeks feeding and all mice were sacrificed immediately (as mentioned above) after the completion of oral glucose tolerance.

In a separate cohort of DIO mice, caecal contents were collected from a high or low responding mouse that was characterized by either high body weight and fasting glycemia or relatively lower body weight and fasting glycemia. Caecal contents of the N and R mice were collected at sacrifice and immediately transplanted to 4 weeks old germfree mice by oral gavage as described above. Then, the NR (n = 16) and RR (n = 14) mice were kept in separate isolators and fed sterilized HF diet and water were provided ad libitum for 10 weeks. Oral glucose tolerance tests were performed at the end of the study before the sacrifice.

For the antibiotic study, 10–12 week old male C57BL/6J mice were fed a gamma-irradiated (25 KGy) high fat diet (diet D12492, Research Diet Inc, New Brunswick) for 10 weeks. For the antibiotic treatment, mice (n = 12/group) were exposed to normal drinking water or a combination of polymyxin B (1.0 g/L) and neomycin (0.5 g/L) in drinking water for 2 weeks. Then, all animals were given water without the antibiotics for 4 weeks. Oral glucose tolerance tests were performed at the end of each period.

### Oral glucose tolerance test

Oral glucose tolerance tests were performed at the end of each study. Mice were fasted for 6 hours during the light cycle before a glucose solution (2 g/kg) was administered by oral gavage. Blood glucose at before (time 0 min) and at 15, 30, 60, and 120 min and plasma insulin before (time 0 min), 15, and 60 min concentrations were analysed from tail vein blood using an Ascensia Elite XL glucometer (Bayer AG, Zurich, Switzerland) and Ultra Sensitive Mouse Insulin ELISA Kit, (Crystal Chem, Downers Grove, IL), respectively. Areas under the glucose and insulin curve were calculated by following trapezoidal rule. Tissue and plasma samples were collected from sacrificed mice post OGTT and stored as described above.

### Measurements of plasma parameters

Blood taken from cardiac puncture at death was collected into chilled EDTA-coated tubes and centrifuged at 1000 g for 10 min. Plasma was aliquoted and frozen at −80 °C until it was analysed. The plasma proinflammatory cytokines IL-1β, IL-6, and TNFα were measured by immunoassay with electrochemiluminescence detection (Meso Scale Discovery, Gaithersburg, MD, USA) according to the manufacturer’s instructions.

### Microbiota analyses

Faecal and caecal contents were collected at sacrifice and the microbiota was analysed by sequencing the V4 region of microbial 16S rDNA. During the time of the study, the technique for microbial sequencing improved significantly, we use 454 pyrosequencing for the NR/RR study and the antibiotics study and Ilumina (Miseq) sequencing for the HF feeding and PO-R and PL-R studies. The primers for 454 sequencing were previously published by Hamdy *et al*.^44^. PCR reactions, PCR conditions and downstream bioinformatics followed the same procedure as Claus *et al*.^45^. In short, the resulting PCR amplicons were sequenced using the GS FLX System. Low quality reads were identified using the strategy of Huse *et al*.^46^ and were removed. Remaining reads were classified using the RDP-Classifier (80% confidence cut-off). As for the samples of HF feeding and PO-R and PL-R studies, DNA was extracted from the faeces of mice. The DNA was quantified and normalized to 50 ng/μl and used for subsequent amplification by following the protocol of Caparaso *et al*.^47^. The resulting PCR products were purified and pooled for sequencing on the MiSeq platform (v2.3). The sequences obtained from an Illumina Miseq sequencer were analysed with MOTHUR (v1.33.3)^48^ and a biome file was created for LEfSe analysis^49^. Ilumina sequence data reported in the paper will be provided on SRA database (SRP075368). Other microbiota data are shown in the Supplementary Tables S1 and S2.

### Gene expression analysis

Total RNA was isolated from frozen epididymal adipose tissue with TRIzol Reagent (Ambion by Life Technologies, Carlsbad, CA) according to the manufacturer’s instructions. Tissue was homogenized with MoBio powerlyzer 24 (Mobio, Carlsbad, CA) and RNA purification was carried out using the QIAcube^®^ (Qiagen, Germany) with the RNeasy^®^ Mini Kit (Qiagen, Germany). The concentration and purity of the RNA samples were determined with the Fragment Analyzer™ (Advanced Analytical Technologies, Inc., Ankeny, USA). Total RNA (1 μg) was reverse transcribed (RT) with qScript cDNA Supermix (Quanta biosciences, Beverly, USA) according to the manufacturer’s protocol. Each cDNA sample was amplified using Lightcycler 1536 DNA Green Master (Roche Diagnostics Ltd., Rotkreuz, Switzerland) on the Lightcycler 480 (Roche Diagnostics Ltd., Rotkreuz, Switzerland) PCR System. Briefly, the reaction conditions consisted of 1.5 μl of cDNA and 0.1 μM primers in a final volume of 10 μl of mix. The following sequences were used for mouse genes: TNFa, forward: ACCGTCAGCCGATTTGCTAT, reverse: TTGACGGCAGAGAGGAGGTT; IL-6, forward: GAAATGATGGATGCTACCAAACTG, reverse: CCAGAAGACCAGAGGAAATTTTCA; IL-1b, forward: CTGCAGCTGGAGAGTGTGGAT, reverse: CAAACCGTTTTTCCATCTTCTTCT; Caspase-1, forward: GAAGGCCCATATAGAGAAAGATTTTATTG, reverse: GACAGGATGTCTCCAAGACACATT. Values were normalized to ribosomal large protein-P0 (RPL-p0) expression; forward: AAAGGAAGAGTCGGAGGAATCAG, reverse: TGGCGGGATTAGTCGAAGAG. The effects of treatments on gene expression were evaluated by calculating the relative expression level as follows: 2 mean Ct genes of interest – mean Ct RPL-p0, using the raw cycle-threshold (Ct) values.

### Statistical analyses of biological data

All biological parameters were tested by unpaired student t-test. Results are presented as mean and standard error of the mean (s.e.m.). All tests were performed two-sided. Linear regression analysis was used to calculate the relationship between the microbiota indices and body weight parameters. Spearman rank correlation analysis was performed between bacterial families and 1^st^ week body weight gain. All analyses were performed with Graphpad Prism 5 (GraphPad Software, Inc. La Jolla, CA). The β-diversity calculation (ThetaYC) of PL-R and PO-R microbiota and analysis of molecular variance (AMOVA) for comparing the two microbiota were carried out in MOTHUR (v1.33.3).

## Additional Information

**How to cite this article**: Rabot, S. *et al*. High fat diet drives obesity regardless the composition of gut microbiota in mice. *Sci. Rep.* **6**, 32484; doi: 10.1038/srep32484 (2016).

## Supplementary Material

Supplementary Information

## Figures and Tables

**Table 1:** Linear regression analysis of microbiota before and after HF feeding to body weight gains and body weights of DIO mice.

	Microbiota before HF feeding				Microbiota after 10 wks HF feeding	
	1^st^ wk body weight gain on HF		Total body weight gain on HF		Final body weight	
	P value	R^2^	P value	R^2^	P value	R^2^
InvSimpson	0.0638	0.08982	0.5237	0.01108	0.0446	0.1074
Shannon	0.0257	0.1274	0.2742	0.03222	0.0301	0.1240
Chao1	0.0497	0.1002	0.1723	0.04974	0.3055	0.02914
F/B	0.2457	0.03527	0.9035	0.0004	0.487	0.0128

Relationships of microbiota diversity with 1^st^ week weight gain, total weight gain and final body weight of conventional mice on a HF diet. Linear regression analysis was used to calculate the three alpha diversity indices, InvSimpson (inverse Simpson), Shannon and Chao 1, and microbiota F/B ratios and different weight gain parameter. Goodness of fit (R^2^) and p-value of correlation are shown.
