# Supplementary material for: High fat diet drives obesity regardless the composition of gut microbiota in mice
Source: Sci Rep. 2016 Aug 31;6:32484. doi: 10.1038/srep32484 (PMC5006052; doi:10.1038/srep32484)
Supplement: Supplementary Information [file srep32484-s1.pdf]

Supplementary information

High fat diet drives obesity regardless the composition of gut microbiota in mice

Sylvie Rabot[1], Mathieu Membrez[2], Florence Blancher[2], Bernard Berger[2], Déborah Moine[2,3], Lutz Krause[2,4], Rodrigo Bibiloni[2,5], Aurélia Bruneau[1], Philippe Gérard[1], Jay Siddharth[3], Christian L. Lauber[3] and Chieh Jason Chou[2,3*]

Supplementary figure S1: Fecal microbiota comparison of PL-R and PO-R mice after one week of HF feeding

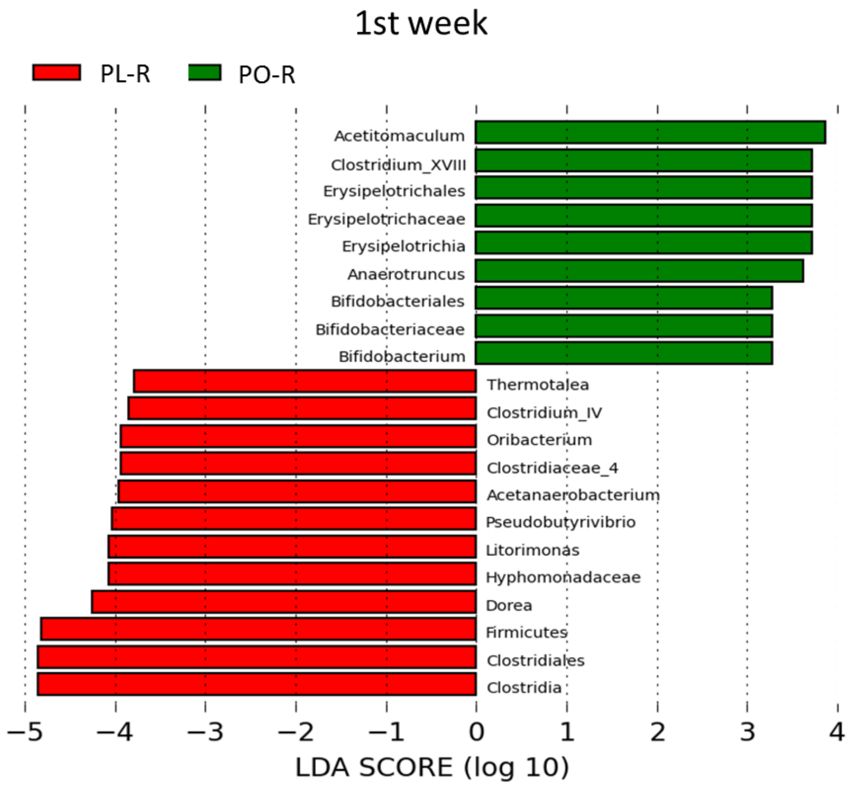

Germfree mice received the transplantation of the PL or PO microbiota and a high fat diet. Fecal samples of PL-R and PO-R mice were collected in 1 week after microbiota transplantation. Comparison of fecal microbiota was performed with LDA effect size (LEfSe). The red area indicates the over-abundance in PL-R microbiota where the green area indicated the over-abundance in PO-R microbiota.

Supplementary figure S2: Fecal microbiota comparison of PL-R and PO-R mice after 10 weeks of HF feeding

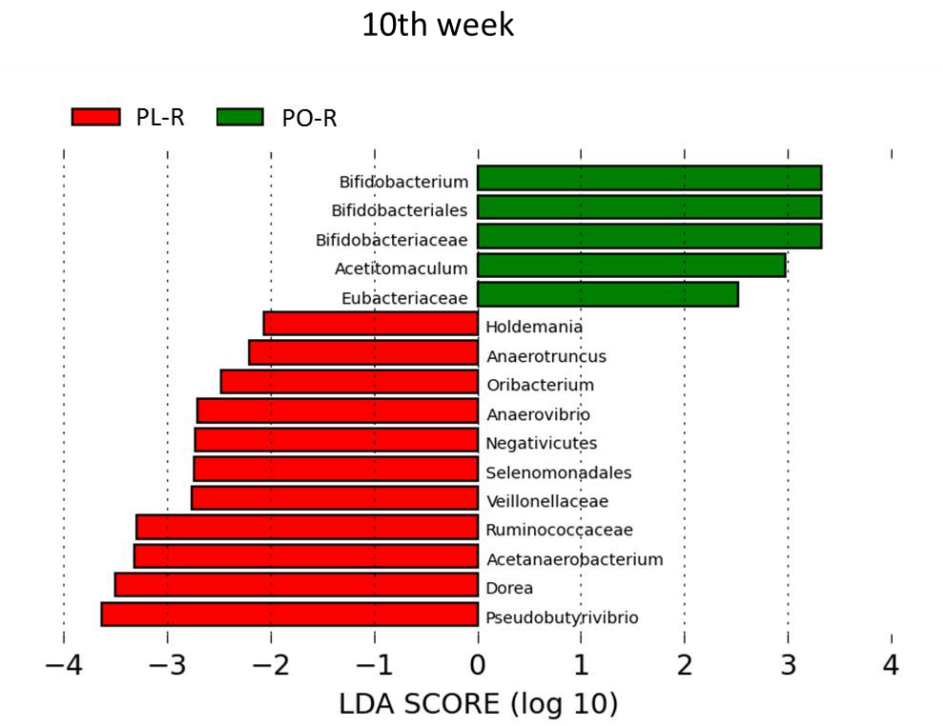

Microbiota composition of PL-R and PO-R mice after 10 weeks HF feeding. Comparison of fecal microbiota was performed with LDA effect size (LEfSe). The red area indicates the over-abundance in PL-R microbiota where the green area indicated the over-abundance in PO-R microbiota.

Supplementary figure S3: Oral glucose tolerance of PL-R and PO-R mice

a

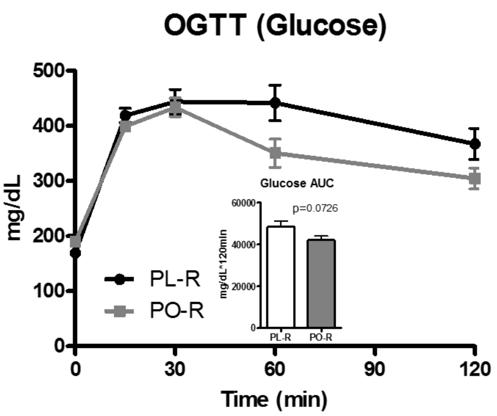

b

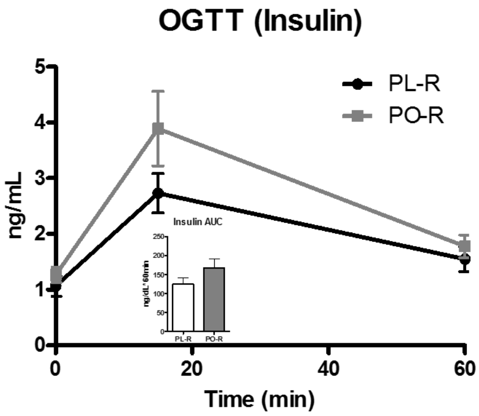

Oral glucose tolerance test was performed after 10 weeks HF feeding. After 6 hours fasting, a bolus of glucose (2 g/kg) was gavaged to PL-R and PO-R mice and glucose (a) and insulin (b) concentrations are shown. The inserts are area under the glucose and insulin curves. Data are mean ± s.e.m., n=12/group. P=0.0726 by unpaired t-test when comparing glucose area under the curve or PL-R and PO-R mice.

Supplementary figure S4: Microbiota composition of the antibiotic treated DIO mice

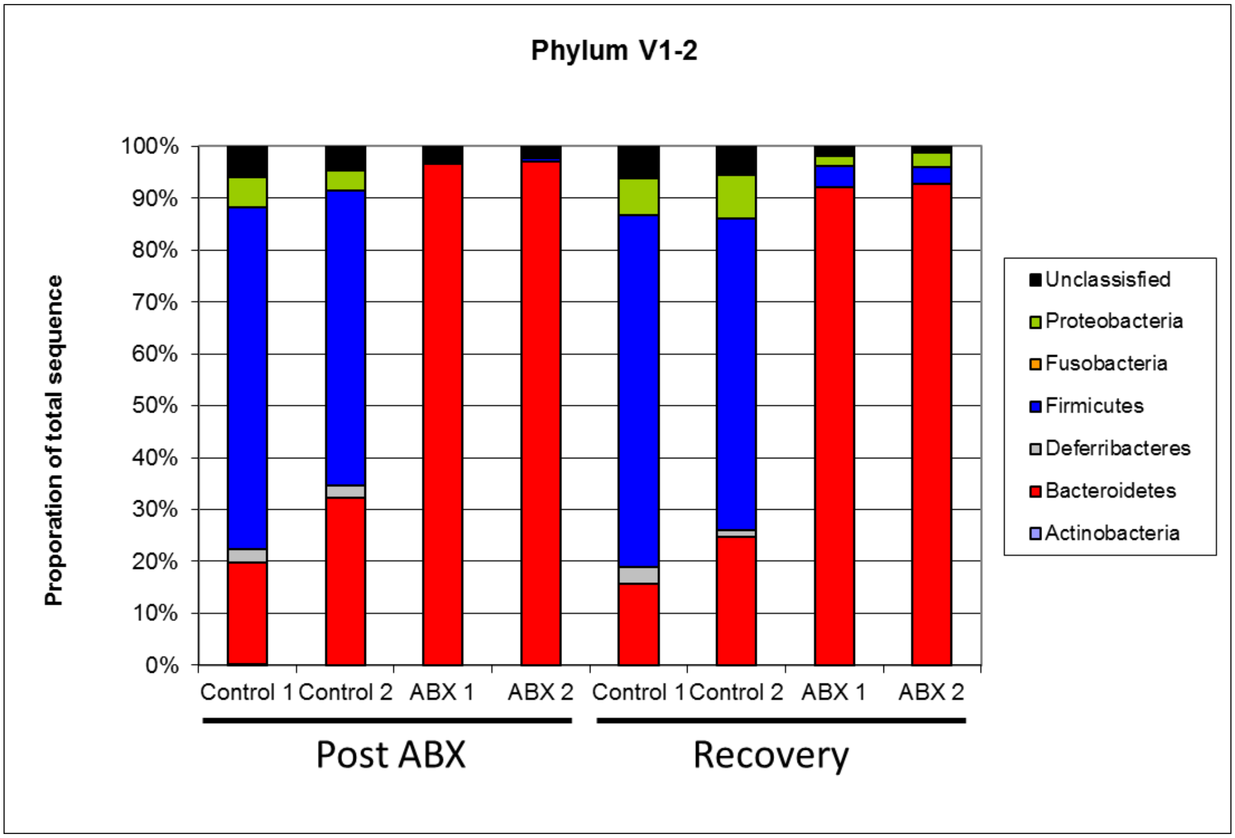

The V1-2 region of bacterial 16S rDNA genes were sequenced and the abundance of bacterial phyla is shown. DIO mice received either a combination of polymyxin B (0.5 g/L) and neomycin (1.0 g/L) in drinking water (ABX) or normal drinking water (Control) for two weeks. Caecal samples were taken from two mice in each group at the end of 2 weeks treatment period (post ABX) and at 4 weeks after the termination of the treatment (Recovery) for microbiota measurement.

Supplementary table S1

Microbiota composition of individual RR and NR mouse.

The procedure for the microbiota analysis is described in Methods. Briefly, DNA samples were extracted from the caecal contents of mice and PCR was performed using primers specific to the V4 region of bacterial 16S rDNA gene. The resulting amplicons were sequenced using the GS FLX System. After removing the low quality reads, remaining sequences were classified using the RDP-Classifier (80% confidence cut-off). Results are presented at phylum, family and genus levels.

Phylum

			V4	V4	V4	V4	V4	V4	V4	V4	V4	V4	V4	V4	V4	V4	V4	V4	V4	V4	V4	V4	V4	V4	V4	V4	V4	V4	V4	V4
Rank	Full	Taxon	NR10	NR11	NR12	NR13	NR14	NR15	NR1	NR2	NR3	NR4	NR5	NR6	NR7	NR8	RR10	RR12	RR13	RR14	RR15	RR16	RR2	RR3	RR4	RR5	RR6	RR7	RR8	RR9
Phylum	Bacteria	Actinobacteria	1	14	12	0	1	4	1	8	16	14	4	25	2	5	4	1	1	6	13	6	5	0	0	6	9	1	1	7
Phylum	Bacteria	Bacteroidetes	240	263	213	85	131	150	35	114	233	866	197	2038	60	189	153	128	156	273	186	536	406	73	84	519	439	157	162	198
Phylum	Bacteria	Deferribacteres	36	52	58	35	76	26	18	36	87	177	41	431	9	34	37	42	63	76	84	150	68	77	15	78	188	28	26	54
Phylum	Bacteria Firmicutes	Firmicutes	731	1111	1136	393	970	545	149	673	902	2560	686	6303	288	572	897	709	748	1321	965	2577	2005	609	371	2178	3210	595	597	931
Phylum	Bacteria	Proteobacteria	24	32	38	23	39	24	5	24	17	134	61	270	25	25	32	25	42	51	21	92	41	23	7	79	114	20	17	53
Phylum	Bacteria	Verrucomicrobia	0	0	0	0	0	0	0	0	9	1	0	0	0	0	0	0	0	0	0	0	0	0	0	0	0	0	0	0
Phylum	Unclassified	Unclassified	3	0	0	1	2	1	0	1	2	2	1	29	0	2	3	0	9	13	5	5	3	0	0	8	20	4	3	1
		total	1035	1472	1457	537	1219	750	208	856	1266	3754	990	9096	384	827	1126	905	1019	1740	1274	3366	2528	782	477	2868	3980	805	806	1244

Family

			V4	V4	V4	V4	V4	V4	V4	V4	V4	V4	V4	V4	V4	V4	V4	V4	V4	V4	V4	V4	V4	V4	V4	V4	V4	V4	V4	V4
Rank	Full	Taxon	NR10	NR11	NR12	NR13	NR14	NR15	NR1	NR2	NR3	NR4	NR5	NR6	NR7	NR8	RR10	RR12	RR13	RR14	RR15	RR16	RR2	RR3	RR4	RR5	RR6	RR7	RR8	RR9
Family	Bacteria Actinobacteria Actinobacteria	Actinobacteria Coriobacteriaceae	1	14	12	0	1	4	1	8	16	14	3	25	2	5	4	1	1	6	13	6	5	0	0	4	9	1	1	6
Family	Bacteria Bacteroidetes	Bacteroidia Bacteroidaceae	41	55	21	15	21	26	12	22	78	282	20	427	12	31	9	7	12	35	15	52	62	17	8	41	31	12	19	9
Family	Bacteria Bacteroidetes Bacteroidia	Bacteroidia Porphyromonadaceae	66	70	94	22	30	43	7	37	50	236	54	719	14	54	40	43	44	93	61	157	139	19	24	174	122	53	48	53
Family	Bacteria Bacteroidetes	Bacteroidia Rikenellaceae	105	103	75	40	73	62	12	41	90	270	109	657	22	85	83	70	85	128	95	277	149	27	44	250	233	78	75	108
Family	Bacteria Deferribacteres Deferribacteres	Deferribacteres Deferribacteraceae	36	52	58	35	76	26	18	36	87	177	41	431	9	34	37	42	63	76	84	150	68	77	15	78	188	28	26	54
Family	Bacteria Firmicutes Bacilli	Bacilli Lactobacillaceae	1	13	1	1	2	2	1	3	10	32	0	34	0	1	1	1	1	1	2	3	40	2	0	4	6	2	5	2
Family	Bacteria Firmicutes Bacilli	Bacilli Streptococcaceae	1	4	0	0	0	0	0	0	1	7	5	19	0	0	0	0	3	1	2	8	6	2	0	0	0	4	3	2
Family	Bacteria Firmicutes Clostridia	Clostridia Incertae Sedis XIV	0	0	0	0	0	0	0	0	0	0	0	2	0	0	0	0	1	0	0	0	1	0	2	0	0	0	0	0
Family	Bacteria Firmicutes Clostridia	Clostridia Lachnospiraceae	535	701	637	253	636	327	107	363	312	1225	379	3748	175	370	600	435	463	942	537	1582	1302	503	222	1487	2347	371	405	511
Family	Bacteria Firmicutes Clostridia Clostridiales	Clostridia Peptostreptococcaceae	0	0	0	1	0	0	0	0	0	7	3	1	0	1	0	1	2	0	0	0	0	0	0	0	2	0	0	0
Family	Bacteria Firmicutes Clostridia	Clostridia Ruminococcaceae	110	158	175	59	132	91	26	106	61	574	135	1062	44	74	124	116	123	135	88	379	288	65	54	290	400	84	89	109
Family	Bacteria Firmicutes Erysipelotrichi Erysipelotrichales	Erysipelotrichi Erysipelotrichaceae	12	127	250	22	105	67	5	162	481	408	98	600	38	73	129	97	85	122	297	399	271	9	59	221	210	87	25	247
Family	Bacteria Proteobacteria Deltaproteobacteria Desulfovibrionales	Deltaproteobacteria Desulfovibrionaceae	22	31	35	15	37	20	5	21	14	122	41	240	14	25	31	22	40	42	20	87	36	21	7	72	100	17	14	52
Family	Bacteria Proteobacteria Gammaproteobacter	Gammaproteobacteria Pasteurellaceae	0	0	0	0	0	0	0	0	0	0	16	0	0	0	0	0	0	0	0	0	1	0	0	0	0	0	2	0
Family	Bacteria Verrucomicrobia Verrucomicrobiae Verrucomicrobiales	Verrucomicrobiae Verrucomicrobiaceae	0	0	0	0	0	0	0	0	9	1	0	0	0	0	0	0	0	0	0	0	0	0	0	0	0	0	0	0
Family	Unclassified	Unclassified	104	140	99	67	106	82	14	55	56	391	80	1113	41	73	67	70	95	155	58	261	152	37	42	240	328	66	91	89
Family	Remaining	Remaining	1	4	0	7	0	0	0	2	1	8	6	18	13	1	1	0	1	4	2	5	8	3	0	7	4	2	3	2
Family	Bacteria Actinobacteria Actinobacteria	Actinobacteria Corynebacteriaceae	0	0	0	0	0	0	0	0	0	0	0	0	0	0	0	0	0	0	0	0	0	0	0	1	0	0	0	0
Family	Bacteria Actinobacteria Actinobacteria	Actinobacteria Micrococcaceae	0	0	0	0	0	0	0	0	0	0	1	0	0	0	0	0	0	0	0	0	0	0	0	0	0	0	0	0
Family	Bacteria Actinobacteria Actinobacteria	Actinobacteria Propionibacteriaceae	0	0	0	0	0	0	0	0	0	0	0	0	0	0	0	0	0	0	0	0	0	0	0	0	0	0	0	0
Family	Bacteria Actinobacteria Actinobacteria	Actinobacteria Bifidobacteriaceae	0	0	0	0	0	0	0	0	0	0	0	0	0	0	0	0	0	0	0	0	0	0	0	1	0	0	0	1
Family	Bacteria Bacteroidetes	Bacteroidia Prevotellaceae	0	0	0	0	0	0	0	0	0	0	0	0	0	0	0	0	0	0	0	0	0	0	0	0	0	0	0	0
Family	Bacteria Bacteroidetes Sphingobacteria Sphingobacteriales	Sphingobacteria Chitinophagaceae	0	0	0	0	0	0	0	0	0	0	0	0	0	0	0	0	0	0	0	0	0	0	0	0	0	0	0	0
Family	Bacteria Firmicutes Bacilli Bacillales	Bacilli Staphylococcaceae	0	0	0	0	0	0	0	0	0	0	0	0	0	0	0	0	0	1	0	0	1	0	0	0	0	0	0	0
Family	Bacteria Firmicutes Bacilli	Bacilli Enterococcaceae	0	0	0	0	0	0	0	1	0	0	0	2	0	0	0	0	0	0	0	0	1	0	0	0	1	0	1	0
Family	Bacteria Firmicutes Clostridia	Clostridia Eubacteriaceae	0	1	0	0	0	0	0	0	0	4	2	5	2	0	0	0	0	1	1	1	1	0	0	1	0	1	1	1
Family	Bacteria Firmicutes Clostridia	Clostridia Incertae Sedis XIII	0	2	0	0	0	0	0	0	0	2	0	6	1	1	1	0	0	0	1	4	2	2	0	3	1	1	0	0
Family	Bacteria Proteobacteria Alphaproteobacteria Alphaproteobacteria	Alphaproteobacteria Alphaproteobacteria_incertae_sedis	0	0	0	0	0	0	0	0	0	0	0	0	0	0	0	0	0	0	0	0	0	0	0	1	0	0	0	0
Family	Bacteria Proteobacteria Alphaproteobacteria	Alphaproteobacteria Bradyrhizobiaceae	0	0	0	0	0	0	0	0	0	0	0	0	0	0	0	0	0	0	0	0	0	0	0	0	0	0	0	0
Family	Bacteria Proteobacteria Betaproteobacteria	Betaproteobacteria Alcaligenaceae	0	0	0	0	0	0	0	0	0	0	0	1	0	0	0	0	0	0	0	0	0	0	0	0	0	0	0	0
Family	Bacteria Proteobacteria Betaproteobacteria	Betaproteobacteria Comamonadaceae	0	0	0	0	0	0	0	0	0	0	0	0	0	0	0	0	0	0	0	0	0	0	0	0	0	0	0	0
Family	Bacteria Proteobacteria Betaproteobacteria	Betaproteobacteria Neisseriaceae	0	0	0	0	0	0	0	0	0	0	0	0	0	0	0	0	0	0	0	0	0	0	0	0	0	0	0	0
Family	Bacteria Proteobacteria Deltaproteobacteria Desulfovibrionales	Deltaproteobacteria Desulfohalobiaceae	0	0	0	0	0	0	0	0	0	1	0	0	0	0	0	0	0	0	0	0	0	0	0	0	1	0	0	0
Family	Bacteria Proteobacteria Gammaproteobacteria Enterobacteriales	Gammaproteobacteria Enterobacteriaceae	0	0	0	7	0	0	0	0	0	1	1	1	0	0	0	0	0	1	0	0	2	0	0	0	0	0	1	0
Family	Bacteria Proteobacteria Gammaproteobacter	Gammaproteobacteria Moraxellaceae	0	1	0	0	0	0	0	0	0	0	1	0	10	0	0	0	0	0	0	0	1	0	0	0	0	0	0	0
Family	Bacteria Proteobacteria Gammaproteobacteria Pseudomonadales	Gammaproteobacteria Pseudomonadaceae	1	0	0	0	0	0	0	1	1	0	0	3	0	0	0	0	1	1	0	0	0	1	0	0	1	0	0	0
Family	Bacteria Proteobacteria Gammaproteobacteria Xanthomonadales	Gammaproteobacteria Xanthomonadaceae	0	0	0	0	0	0	0	0	0	0	1	0	0	0	0	0	0	0	0	0	0	0	0	0	0	0	0	0

	V4	V4	V4	V4	V4	V4	V4	V4	V4	V4	V4	V4	V4	V4	V4	V4	V4	V4	V4	V4	V4	V4	V4	V4	V4	V4	V4	V4
Taxon	NR10	NR11	NR12	NR13	NR14	NR15	NR1	NR2	NR3	NR4	NR5	NR6	NR7	NR8	RR10	RR12	RR13	RR14	RR15	RR16	RR2	RR3	RR4	RR5	RR6	RR7	RR8	RR9
Actinobacteria Actinobacteria	1	14	12	0	1	4	1	8	16	14	4	25	2	5	4	1	1	6	13	6	5	0	0	6	9	1	1	7
Bacteroidia	213	228	190	77	124	133	31	100	218	789	183	1814	48	170	132	120	143	257	171	486	350	63	76	467	386	143	142	171
Sphingobacteria Sphingobacteria	0	0	0	0	0	0	0	0	0	0	0	0	0	0	0	0	0	0	0	0	0	0	0	0	0	0	0	0
Deferribacteres Deferribacteres	36	52	58	35	76	26	18	36	87	177	41	431	9	34	37	42	63	76	84	150	68	77	15	78	188	28	26	54
Bacilli Bacilli	2	18	1	1	2	2	1	4	11	39	5	56	0	1	1	1	4	3	4	11	48	4	0	4	7	6	9	4
Clostridia	717	965	881	370	859	476	143	507	409	2113	582	5637	250	498	766	611	659	1193	660	2163	1685	596	311	1952	2993	502	562	678
Erysipelotrichi	12	127	250	22	105	67	5	162	481	408	98	600	38	73	129	97	85	122	297	399	271	9	59	221	210	87	25	247
Alphaproteobacteria	0	0	1	0	0	0	0	0	0	0	0	4	0	0	0	1	0	0	0	0	0	0	0	2	1	0	0	0
Betaproteobacteria	0	0	0	0	0	0	0	0	0	0	0	1	0	0	0	0	0	0	0	0	0	0	0	0	0	0	0	0
Deltaproteobacteria	23	31	37	16	39	24	5	23	16	133	42	260	15	25	32	24	41	49	21	91	37	22	7	77	112	20	14	53
Gammaproteobacteria	1	1	0	7	0	0	0	1	1	1	19	4	10	0	0	0	1	2	0	0	4	1	0	0	1	0	3	0
Verrucomicrobiae Verrucomicrobiae	0	0	0	0	0	0	0	0	9	1	0	0	0	0	0	0	0	0	0	0	0	0	0	0	0	0	0	0
Unclassified	30	36	27	9	13	18	4	15	18	79	16	264	12	21	25	8	22	32	24	60	60	10	9	61	73	18	24	30

Genus

			V4	V4	V4	V4	V4	V4	V4	V4	V4	V4	V4	V4	V4	V4	V4	V4	V4	V4	V4	V4	V4	V4	V4	V4	V4	V4	V4	V4
Rank	Full	Taxon	NR10	NR11	NR12	NR13	NR14	NR15	NR1	NR2	NR3	NR4	NR5	NR6	NR7	NR8	RR10	RR12	RR13	RR14	RR15	RR16	RR2	RR3	RR4	RR5	RR6	RR7	RR8	RR9
Genus	Bacteria Bacteroidetes Bacteroidia	Bacteroidia Bacteroides	41	55	21	15	21	26	12	22	78	282	20	427	12	31	9	7	12	35	15	52	62	17	8	41	31	12	19	9
Genus	Bacteria Bacteroidetes Bacteroidia	Bacteroidia Barnesiella	40	48	52	12	10	27	4	29	27	117	23	353	7	29	15	22	23	47	32	67	57	9	15	98	52	29	20	27
Genus	Bacteria Bacteroidetes Bacteroidia	Bacteroidia Odoribacter	6	9	17	4	9	8	0	2	7	32	13	120	4	16	14	7	11	12	8	34	36	4	3	29	34	11	11	12
Genus	Bacteria Bacteroidetes Bacteroidia Bacteroidales	Bacteroidia Parabacteroides	1	2	2	0	2	1	1	1	4	23	6	64	0	0	0	2	2	6	1	8	8	3	0	14	7	2	1	1
Genus	Bacteria Bacteroidetes Bacteroidia	Bacteroidia Alistipes	105	103	75	40	72	62	12	41	90	268	109	656	22	85	83	70	85	128	95	277	149	27	44	250	233	78	75	108
Genus	Bacteria Deferribacteres Deferribacteres	Deferribacteres Mucispirillum	36	52	58	35	76	26	18	36	87	177	41	431	9	34	37	42	63	76	84	150	68	77	15	78	188	28	26	54
Genus	Bacteria Firmicutes Bacilli Lactobacillales	Bacilli Lactobacillus	1	13	1	1	2	2	1	3	10	32	0	34	0	1	1	1	1	1	2	3	40	2	0	4	6	2	5	2
Genus	Bacteria Firmicutes Clostridia Clostridiales	Clostridia Dorea	29	59	59	30	46	34	19	55	35	151	56	363	20	38	46	33	34	106	60	161	133	62	27	106	126	28	31	64
Genus	Bacteria Firmicutes Clostridia Clostridiales	Clostridia Robinsoniella	0	0	0	0	0	0	0	0	0	1	0	0	0	0	0	6	28	44	13	66	1	0	26	20	1	20	25	50
Genus	Bacteria Firmicutes Clostridia Clostridiales	Clostridia Roseburia	27	16	28	4	56	26	10	17	15	69	5	39	1	7	22	2	22	41	36	72	22	31	14	97	95	4	7	16
Genus	Bacteria Firmicutes Clostridia Clostridiales	Clostridia Hydrogenoanaerobacterium	8	13	9	7	19	20	4	16	6	70	11	105	5	5	10	9	9	8	13	21	18	9	1	16	37	6	10	6
Genus	Bacteria Firmicutes Clostridia Clostridiales	Clostridia Oscillibacter	19	20	23	7	33	13	1	14	12	88	27	160	5	11	16	26	16	21	12	53	33	4	9	38	56	15	10	19
Genus	Bacteria Firmicutes Erysipelotrichi Erysipelotrichales	Erysipelotrichi Allobaculum	9	123	245	22	104	67	5	160	478	407	90	587	36	71	126	92	84	120	294	396	269	9	59	220	207	87	25	244
Genus	Bacteria Proteobacteria Deltaproteobacteria Desulfovibrionales	Deltaproteobacteria Desulfovibrio	22	30	34	15	34	19	4	21	14	121	39	233	14	24	30	22	40	42	20	82	35	20	6	69	94	16	13	49
Genus	Bacteria Proteobacteria Deltaproteobacteria Desulfovibrionales	Deltaproteobacteria Lawsonia	0	0	0	0	0	0	0	0	0	0	0	0	0	0	0	0	0	0	0	0	0	0	0	0	0	0	0	0
Genus	Unclassified	Unclassified	668	873	777	329	709	397	112	404	359	1777	501	5218	219	456	664	540	545	981	548	1759	1473	476	239	1680	2649	438	480	547
Genus	Remaining	Remaining	23	56	56	16	26	22	5	35	44	139	49	306	30	19	53	24	44	72	41	165	124	32	11	108	164	29	48	36
Genus	Bacteria Actinobacteria Actinobacteria Actinomycetales	Actinobacteria Corynebacterium	0	0	0	0	0	0	0	0	0	0	0	0	0	0	0	0	0	0	0	0	0	0	0	1	0	0	0	0
Genus	Bacteria Actinobacteria Actinobacteria	Actinobacteria Rothia	0	0	0	0	0	0	0	0	0	0	1	0	0	0	0	0	0	0	0	0	0	0	0	0	0	0	0	0
Genus	Bacteria Actinobacteria Actinobacteria Actinomycetales	Actinobacteria Propionibacterium	0	0	0	0	0	0	0	0	0	0	0	0	0	0	0	0	0	0	0	0	0	0	0	0	0	0	0	0
Genus	Bacteria Actinobacteria Actinobacteria Bifidobacteriales	Actinobacteria Bifidobacterium	0	0	0	0	0	0	0	0	0	0	0	0	0	0	0	0	0	0	0	0	0	0	0	1	0	0	0	1
Genus	Bacteria Actinobacteria Actinobacteria	Actinobacteria Asaccharobacter	0	0	0	0	0	0	0	0	0	0	0	0	0	0	0	0	0	0	0	0	0	0	0	0	0	0	0	0
Genus	Bacteria Actinobacteria Actinobacteria	Actinobacteria Enterorhabdus	1	3	3	0	1	4	1	3	5	9	3	23	2	2	2	1	1	6	5	6	4	0	0	3	8	1	1	3
Genus	Bacteria Actinobacteria Actinobacteria	Actinobacteria Olsenella	0	11	9	0	0	0	0	5	10	5	0	1	0	2	2	0	0	0	8	0	0	0	0	0	0	0	0	3
Genus	Bacteria Bacteroidetes Bacteroidia Bacteroidales	Bacteroidia Butyricimonas	2	0	1	0	0	1	0	0	1	3	0	5	0	0	0	0	0	0	0	3	0	0	0	1	0	0	0	0
Genus	Bacteria Bacteroidetes Bacteroidia	Bacteroidia Paludibacter	0	0	0	0	0	0	0	0	0	0	0	0	0	0	0	0	0	0	0	0	0	0	0	0	0	0	0	0
Genus	Bacteria Bacteroidetes Bacteroidia	Bacteroidia Rikenella	0	0	0	0	0	0	0	0	0	1	0	0	0	0	0	0	0	0	0	0	0	0	0	0	0	0	0	0
Genus	Bacteria Bacteroidetes Sphingobacteria Sphingobacteriales Chitinophagaceae	Sphingobacteria Sediminibacterium	0	0	0	0	0	0	0	0	0	0	0	0	0	0	0	0	0	0	0	0	0	0	0	0	0	0	0	0
Genus	Bacteria Firmicutes Bacilli Bacillales Staphylococcaceae	Bacilli Staphylococcus	0	0	0	0	0	0	0	0	0	0	0	0	0	0	0	0	0	1	0	0	1	0	0	0	0	0	0	0
Genus	Bacteria Firmicutes Bacilli Lactobacillales	Bacilli Enterococcus	0	0	0	0	0	0	0	1	0	0	0	2	0	0	0	0	0	0	0	0	1	0	0	0	1	0	1	0
Genus	Bacteria Firmicutes Bacilli Lactobacillales	Bacilli Lactococcus	1	4	0	0	0	0	0	0	1	7	5	18	0	0	0	0	3	1	2	8	6	2	0	0	0	4	1	2
Genus	Bacteria Firmicutes Bacilli Lactobacillales	Bacilli Streptococcus	0	0	0	0	0	0	0	0	0	0	0	1	0	0	0	0	0	0	0	0	0	0	0	0	0	0	2	0

Genus	Bacteria Firmicutes Clostridia Clostridiales	Clostridia Anaerofustis	0	0	0	0	0	0	0	0	0	0	0	0	1	0	0	0	0	0	0	0	0	0	0	0	0	0	0	0
Genus	Bacteria Firmicutes Clostridia Clostridiales	Clostridia Eubacterium	0	1	0	0	0	0	0	0	0	4	2	4	1	0	0	0	0	1	1	1	1	0	0	1	0	1	1	1
Genus	Bacteria Firmicutes Clostridia Clostridiales	Clostridia Anaerovorax	0	2	0	0	0	0	0	0	0	2	0	6	1	1	1	0	0	0	1	4	2	2	0	3	1	1	0	0
Genus	Bacteria Firmicutes Clostridia Clostridiales	Clostridia Blautia	0	0	0	0	0	0	0	0	0	0	0	2	0	0	0	0	1	0	0	0	1	0	2	0	0	0	0	0
Genus	Bacteria Firmicutes Clostridia Clostridiales	Clostridia Anaerosporobacter	2	1	5	3	3	0	0	2	0	10	1	24	1	1	5	0	1	2	2	6	4	1	0	6	8	1	6	1
Genus	Bacteria Firmicutes Clostridia Clostridiales	Clostridia Anaerostipes	0	0	0	0	0	0	0	0	0	0	0	0	0	0	0	0	0	0	0	0	0	0	0	0	0	0	0	0
Genus	Bacteria Firmicutes Clostridia Clostridiales	Clostridia Coprococcus	0	0	0	0	0	0	0	0	0	0	0	0	0	0	0	0	0	0	0	0	0	0	0	0	1	0	0	0
Genus	Bacteria Firmicutes Clostridia Clostridiales	Clostridia Hespellia	0	0	0	0	0	0	0	0	0	0	0	0	0	0	0	0	0	0	0	0	0	0	0	0	0	0	0	0
Genus	Bacteria Firmicutes Clostridia Clostridiales	Clostridia Johnsonella	3	12	11	0	7	8	1	8	5	23	8	50	4	4	12	8	11	19	4	23	23	13	4	38	57	9	7	12
Genus	Bacteria Firmicutes Clostridia Clostridiales	Clostridia Lachnobacterium	0	0	0	0	0	0	0	0	0	0	0	0	0	0	0	0	0	0	0	0	0	0	0	0	0	0	0	0
Genus	Bacteria Firmicutes Clostridia Clostridiales	Clostridia Marvinbryantia	2	0	4	0	6	0	0	1	3	6	0	32	3	2	3	0	4	3	0	18	1	0	0	0	1	0	4	0
Genus	Bacteria Firmicutes Clostridia Clostridiales	Clostridia Parasporobacterium	0	0	0	0	0	0	0	0	0	0	0	0	0	0	2	1	0	1	1	8	9	1	0	9	4	0	1	1
Genus	Bacteria Firmicutes Clostridia Clostridiales	Clostridia Pseudobutyrivibrio	1	0	2	0	0	0	0	0	0	0	0	3	0	0	0	0	0	2	3	1	0	0	0	0	1	0	0	0
Genus	Bacteria Firmicutes Clostridia Clostridiales	Clostridia Syntrophococcus	0	0	0	0	0	0	0	0	0	0	0	0	0	0	0	0	0	0	0	0	0	0	0	0	1	0	0	0
Genus	Bacteria Firmicutes Clostridia Clostridiales Peptostreptococcac	Clostridia Peptostreptococcus	0	0	0	0	0	0	0	0	0	0	0	0	0	0	0	0	0	0	0	0	0	0	0	0	0	0	0	0
Genus	Bacteria Firmicutes Clostridia Clostridiales Peptostreptococcac	Clostridia Sporacetigenium	0	0	0	0	0	0	0	0	0	0	0	0	0	0	0	0	0	0	0	0	0	0	0	0	0	0	0	0
Genus	Bacteria Firmicutes Clostridia Clostridiales	Clostridia Acetanaerobacterium	0	0	0	0	0	0	0	0	0	0	0	8	0	0	0	0	0	0	0	0	0	0	0	0	1	0	0	0
Genus	Bacteria Firmicutes Clostridia Clostridiales	Clostridia Acetivibrio	0	0	0	0	0	0	1	0	0	1	0	2	0	0	0	1	0	0	0	0	0	0	0	1	0	0	1	0
Genus	Bacteria Firmicutes Clostridia Clostridiales	Clostridia Anaerotruncus	2	2	4	1	3	4	0	4	2	20	1	32	0	1	1	3	1	8	3	13	3	2	2	8	8	3	1	3
Genus	Bacteria Firmicutes Clostridia Clostridiales	Clostridia Butyricicoccus	0	2	2	0	0	1	0	0	0	0	1	2	1	2	0	0	0	3	0	0	2	0	0	1	0	0	1	0
Genus	Bacteria Firmicutes Clostridia Clostridiales	Clostridia Ethanoligenens	0	0	0	0	0	0	0	0	0	0	0	0	0	0	0	0	0	0	0	0	0	0	0	1	0	0	0	0
Genus	Bacteria Firmicutes Clostridia Clostridiales	Clostridia Lactonifactor	0	0	0	0	0	0	0	0	0	0	0	0	0	0	0	0	0	0	0	0	0	0	0	0	0	0	0	0
Genus	Bacteria Firmicutes Clostridia Clostridiales	Clostridia Papillibacter	3	6	3	1	0	0	0	1	3	13	2	16	2	0	3	0	2	0	0	9	3	0	0	2	8	3	0	0
Genus	Bacteria Firmicutes Clostridia Clostridiales	Clostridia Ruminococcus	0	4	3	1	4	4	0	2	2	13	1	35	3	2	3	0	0	4	0	19	0	0	1	0	4	3	2	2
Genus	Bacteria Firmicutes Clostridia Clostridiales	Clostridia Sporobacter	5	7	9	3	2	0	2	7	2	20	5	34	1	2	19	10	19	19	11	46	59	10	2	32	58	3	16	7
Genus	Bacteria Firmicutes Clostridia Clostridiales	Clostridia Subdoligranulum	0	0	0	0	0	0	0	0	0	0	0	0	0	0	0	0	0	0	0	0	0	0	0	0	0	0	0	0
Genus	Bacteria Firmicutes Erysipelotrichi Erysipelotrichales	Erysipelotrichi Coprobacillus	0	0	0	0	0	0	0	0	0	0	0	0	0	0	0	0	0	0	0	0	0	0	0	0	1	0	0	0
Genus	Bacteria Firmicutes Erysipelotrichi Erysipelotrichales	Erysipelotrichi Holdemania	0	0	0	0	0	0	0	0	0	0	0	1	0	0	0	0	0	0	0	0	0	0	0	0	0	0	0	0
Genus	Bacteria Firmicutes Erysipelotrichi Erysipelotrichales	Erysipelotrichi Solobacterium	0	0	0	0	0	0	0	0	0	0	0	0	0	0	0	0	0	0	0	0	0	0	0	0	0	0	0	0

Genus	Bacteria<br>Proteobacteria<br>Alphaproteobacteria	Alphaproteobacter<br>ia Bradyrhizobium	0	0	0	0	0	0	0	0	0	0	0	0	0	0	0	0	0	0	0	0	0	0	0	0	0	0	0	0
Genus	Bacteria<br>Proteobacteria<br>Betaproteobacteria<br>Burkholderiales	Betaproteobacteri<br>a Parasutterella	0	0	0	0	0	0	0	0	0	0	0	1	0	0	0	0	0	0	0	0	0	0	0	0	0	0	0	0
Genus	Bacteria<br>Proteobacteria<br>Betaproteobacteria<br>Burkholderiales	Betaproteobacteri<br>a Pelomonas	0	0	0	0	0	0	0	0	0	0	0	0	0	0	0	0	0	0	0	0	0	0	0	0	0	0	0	0
Genus	Bacteria<br>Proteobacteria<br>Deltaproteobacteria<br>Desulfovibrionales<br>Desulfovibrionaceae	Deltaproteobacteri<br>a Desulfocurvus	0	0	0	0	0	0	0	0	0	0	0	0	0	0	0	0	0	0	0	0	0	0	0	0	0	0	0	0
Genus	Bacteria<br>Proteobacteria<br>Gammaproteobacter<br>ia Enterobacteriales	Gammaproteobact<br>eria Enterobacter	0	0	0	0	0	0	0	0	0	0	0	0	0	0	0	0	0	0	0	0	0	0	0	0	0	0	0	0
Genus	Bacteria<br>Proteobacteria<br>Gammaproteobacter<br>ia Enterobacteriales<br>Enterobacteriaceae	Gammaproteobact<br>eria<br>Escherichia/Shigell<br>a	0	0	0	7	0	0	0	0	0	1	1	1	0	0	0	0	0	1	0	0	2	0	0	0	0	0	1	0
Genus	Bacteria<br>Proteobacteria<br>Gammaproteobacter<br>ia Pasteurellales	Gammaproteobact<br>eria Haemophilus	0	0	0	0	0	0	0	0	0	0	16	0	0	0	0	0	0	0	0	0	1	0	0	0	0	0	2	0
Genus	Bacteria<br>Proteobacteria<br>Gammaproteobacter<br>ia Pseudomonadales	Gammaproteobact<br>eria Acinetobacter	0	0	0	0	0	0	0	0	0	0	0	0	0	0	0	0	0	0	0	0	0	0	0	0	0	0	0	0
Genus	Bacteria<br>Proteobacteria<br>Gammaproteobacter<br>ia Pseudomonadales	Gammaproteobact<br>eria Alkanindiges	0	0	0	0	0	0	0	0	0	0	0	0	0	0	0	0	0	0	0	0	0	0	0	0	0	0	0	0
Genus	Bacteria<br>Proteobacteria<br>Gammaproteobacter<br>ia Pseudomonadales	Gammaproteobact<br>eria Enhydrobacter	0	0	0	0	0	0	0	0	0	0	0	0	0	0	0	0	0	0	0	0	0	0	0	0	0	0	0	0
Genus	Bacteria<br>Proteobacteria<br>Gammaproteobacter<br>ia Pseudomonadales	Gammaproteobact<br>eria Moraxella	0	1	0	0	0	0	0	0	0	0	1	0	10	0	0	0	0	0	0	0	1	0	0	0	0	0	0	0
Genus	Bacteria<br>Proteobacteria<br>Gammaproteobacter<br>ia Pseudomonadales	Gammaproteobact<br>eria Pseudomonas	1	0	0	0	0	0	0	1	1	0	0	3	0	0	0	0	1	1	0	0	0	1	0	0	1	0	0	0
Genus	Bacteria<br>Proteobacteria<br>Gammaproteobacter<br>ia Xanthomonadales<br>Xanthomonadaceae	Gammaproteobact<br>eria<br>Stenotrophomonas	0	0	0	0	0	0	0	0	0	0	1	0	0	0	0	0	0	0	0	0	0	0	0	0	0	0	0	0
Genus	Bacteria<br>Verrucomicrobia<br>Verrucomicrobiae<br>Verrucomicrobiales	Verrucomicrobiae<br>Akkermansia	0	0	0	0	0	0	0	0	9	1	0	0	0	0	0	0	0	0	0	0	0	0	0	0	0	0	0	0

Supplementary table S2

Microbiota composition of antibiotics treated and untreated mice.

Diet-induced obesity mice were treated with a mixture of polymyxin B and neomycin in drinking water for 2 weeks. Mice were sacrificed at the end of treatment and caecal contents were used for DNA extraction (post ABX group). In parallel, another group of mice was kept for additional 4 weeks without antibiotics (Recovery group) and their caecal contents were harvest for the DNA preparation. PCR was performed using primers specific to the V1-2 region of bacterial 16S rDNA gene. The resulting amplicons were sequenced using the GS FLX System. After removing the low quality reads, remaining sequences were classified using the RDP-Classifier (80% confidence cut-off). Results are presented at phylum, family and genus levels.

RDP Classifications										
			V12	V12	V12	V12	V12	V12	V12	V12
			Post ABX				Recovery			
Rank	Full	Taxon	Control 1	Control 2	ABX 1	ABX 2	Control 1	Control 2	ABX 1	ABX 2
Phylum	Root Bacteria Actinobacteria	Actinobacteria	5	0	0	0	0	0	1	0
Phylum	Root Bacteria Bacteroidetes	Bacteroidetes	477	509	2065	1971	229	578	1970	1814
Phylum	Root Bacteria Deferribacteres	Deferribacteres	65	36	0	0	49	26	0	0
Phylum	Root Bacteria Firmicutes	Firmicutes	1606	896	6	13	996	1401	85	64
Phylum	Root Bacteria Fusobacteria	Fusobacteria	0	0	0	0	0	0	0	0
Phylum	Root Bacteria Proteobacteria	Proteobacteria	140	63	0	0	106	194	41	52
Phylum	Unclassified	Unclassified	146	72	64	45	89	129	41	25
		Total	2439	1576	2135	2029	1469	2328	2138	1955

			V12	V12	V12	V12	V12	V12	V12	V12
			Post ABX				Recovery			
Rank	Full	Taxon	Control 1	Control 2	ABX 1	ABX 2	Control 1	Control 2	ABX 1	ABX 2
Genus	Root Bacteria Actinobacteria Actinobacteria Bifidobacteriales Bifidobacteriaceae Bifidobacterium	Actinobacteria Bifidobacterium	4	0	0	0	0	0	0	0
Genus	Root Bacteria Bacteroidetes Bacteroidetes Bacteroidales Bacteroidaceae Bacteroides	Bacteroidetes Bacteroides	104	275	1835	1724	55	189	1510	1557
Genus	Root Bacteria Bacteroidetes Bacteroidetes Bacteroidales Porphyromonadaceae Parabacteroides	Bacteroidetes Parabacteroides	8	15	103	165	5	14	225	163
Genus	Root Bacteria Bacteroidetes Bacteroidetes Bacteroidales Prevotellaceae Prevotella	Bacteroidetes Prevotella	0	0	0	0	0	0	1	0
Genus	Root Bacteria Bacteroidetes Bacteroidetes Bacteroidales Rikenellaceae Alistipes	Bacteroidetes Alistipes	199	124	103	61	112	260	211	84
Genus	Root Bacteria Deferribacteres Deferribacteres Deferribacterales Deferribacteraceae Mucispirillum	Deferribacteres Mucispirillum	65	36	0	0	49	26	0	0
Genus	Root Bacteria Firmicutes Bacilli Bacillales Staphylococcaceae Staphylococcus	Firmicutes Staphylococcus	0	0	0	0	0	0	0	0
Genus	Root Bacteria Firmicutes Bacilli Lactobacillales Enterococcaceae Enterococcus	Firmicutes Enterococcus	0	0	1	2	0	0	0	2
Genus	Root Bacteria Firmicutes Bacilli Lactobacillales Lactobacillaceae Lactobacillus	Firmicutes Lactobacillus	1	6	1	0	1	1	3	0
Genus	Root Bacteria Firmicutes Bacilli Lactobacillales Leuconostocaceae Weissella	Firmicutes Weissella	1	0	0	0	0	0	0	0
Genus	Root Bacteria Firmicutes Bacilli Lactobacillales Streptococcaceae Lactococcus	Firmicutes Lactococcus	1	0	0	6	3	1	4	19
Genus	Root Bacteria Firmicutes Bacilli Lactobacillales Streptococcaceae Streptococcus	Firmicutes Streptococcus	0	0	0	0	0	0	0	0
Genus	Root Bacteria Firmicutes Clostridia Clostridiales IncertaeSedisXIII Anaerovorax	Firmicutes Anaerovorax	4	0	0	0	3	1	0	0
Genus	Root Bacteria Firmicutes Clostridia Clostridiales Lachnospiraceae Anaerostipes	Firmicutes Anaerostipes	0	0	2	0	0	0	2	0
Genus	Root Bacteria Firmicutes Clostridia Clostridiales Lachnospiraceae Butyrivibrio	Firmicutes Butyrivibrio	1	0	0	0	0	0	0	0
Genus	Root Bacteria Firmicutes Clostridia Clostridiales Lachnospiraceae Dorea	Firmicutes Dorea	1	0	0	0	0	0	0	0
Genus	Root Bacteria Firmicutes Clostridia Clostridiales Lachnospiraceae Johnsonella	Firmicutes Johnsonella	0	0	0	0	0	0	0	0
Genus	Root Bacteria Firmicutes Clostridia Clostridiales Lachnospiraceae Roseburia	Firmicutes Roseburia	1	1	0	0	34	0	0	0
Genus	Root Bacteria Firmicutes Clostridia Clostridiales Ruminococcaceae Anaerotruncus	Firmicutes Anaerotruncus	0	1	0	0	3	6	0	0
Genus	Root Bacteria Firmicutes Clostridia Clostridiales Ruminococcaceae Faecalibacterium	Firmicutes Faecalibacterium	3	0	0	0	0	0	0	0
Genus	Root Bacteria Firmicutes Clostridia Clostridiales Ruminococcaceae Sporobacter	Firmicutes Sporobacter	0	0	0	0	0	0	0	0
Genus	Root Bacteria Firmicutes Clostridia Clostridiales Ruminococcaceae Subdoligranulum	Firmicutes Subdoligranulum	1	0	0	0	0	0	0	0
Genus	Root Bacteria Firmicutes Clostridia Clostridiales Veillonellaceae Megasphaera	Firmicutes Megasphaera	0	0	0	0	0	1	0	0
Genus	Root Bacteria Firmicutes Erysipelotrichi Erysipelotrichales Erysipelotrichaceae Allobaculum	Firmicutes Allobaculum	0	0	0	0	0	0	0	0
Genus	Root Bacteria Firmicutes Erysipelotrichi Erysipelotrichales Erysipelotrichaceae Catenibacterium	Firmicutes Catenibacterium	0	0	0	0	0	1	0	0
Genus	Root Bacteria Fusobacteria Fusobacteria Fusobacteriales Fusobacteriaceae Fusobacterium	Fusobacteria Fusobacterium	0	0	0	0	0	0	0	0
Genus	Root Bacteria Proteobacteria Deltaproteobacteria Desulfovibrionales Desulfovibrionaceae Bilophila	Proteobacteria Bilophila	132	57	0	0	101	183	0	0
Genus	Root Bacteria Proteobacteria Gammaproteobacteria Enterobacteriales Enterobacteriaceae Citrobacter	Proteobacteria Citrobacter	0	0	0	0	0	0	0	1
Genus	Root Bacteria Proteobacteria Gammaproteobacteria Enterobacteriales Enterobacteriaceae Enterobacter	Proteobacteria Enterobacter	0	0	0	0	0	0	4	2
Genus	Root Bacteria Proteobacteria Gammaproteobacteria Enterobacteriales Enterobacteriaceae Klebsiella	Proteobacteria Klebsiella	0	0	0	0	0	0	0	0
Genus	Root Bacteria Proteobacteria Gammaproteobacteria Enterobacteriales Enterobacteriaceae Shigella	Proteobacteria Shigella	0	0	0	0	0	0	0	0
Genus	Unclassified	Unclassified	1913	1061	90	71	1103	1645	178	127
		Total	2439	1576	2135	2029	1469	2328	2138	1955
